# Bulk Modification of Poly(lactide) (PLA) via Copolymerization with Poly(propylene glycol) Diglycidylether (PPGDGE)

**DOI:** 10.3390/polym10111184

**Published:** 2018-10-24

**Authors:** Sandra Castillejos, Jorge Cerna, Francisco Meléndez, María Eugenia Castro, Rocío Aguilar, César Márquez-Beltrán, Maykel González

**Affiliations:** 1 Facultad de Ciencias Químicas, Benemérita Universidad Autónoma de Puebla, Ciudad Universitaria, Puebla 72570, Mexico; sandra.castillejos@alumno.buap.mx (S.C.); francisco.melendez@correo.buap.mx (F.M.); raguilar@ifuap.buap.mx (R.A.); 2 Instituto de Ciencias de la Universidad Autónoma de Puebla, Benemérita Universidad Autónoma de Puebla, Ciudad Universitaria, Puebla 72570, Mexico; mareug.castro@correo.buap.mx; 3 Instituto de Física, Benemérita Universidad Autónoma de Puebla, Ciudad Universitaria, Puebla 72570, Mexico; cmarquez@ifuap.buap.mx; 4 Tecnológico de Monterrey, Escuela de Ingeniería y Ciencias, Campus Puebla, Puebla 72453, Mexico; mikegcu@gmail.com

**Keywords:** Poly(lactide), copolymerization, PPGDGE, contact angle, hydrophilic

## Abstract

Copolymers of l-lactide and poly(propylene glycol) diglycidyl ether (PPGDGE_380_) were synthesized by ring opening polymerization (ROP). Stannous octoate was used as the catalyst and 1-dodecanol as the initiator. The effect of the variables on the thermal properties of the copolymers was investigated by differential scanning calorimetry (DSC). Contact angle measurements were made in order to study the wettability of the synthesized copolymers. The copolymers differed widely in their physical characteristics, ranging from weak elastomers to tougher thermoplastics, according to the ratio of l-lactide and PPGDGE_380_. The results showed that the copolymers were more hydrophilic than neat Poly(lactide) (PLA) and the monomer ratio had a strong influence on the hydrophilic properties.

## 1. Introduction

Recently, the increasing emphasis on sustainability in the polymer industry has become a necessity and the reason why great efforts have been made to replace existing polymers with biodegradable and biorenewable alternatives [1,2,3,4,5]. The most representative thermoplastic in this area is polylactic acid (PLA), a bio-based polyester derived from lactic acid or lactide that is biodegradable, recyclable and compostable [6,7,8].

The PLA homopolymer has physical properties that make it suitable for a wide range of applications. It has good mechanical properties, thermal stability, transparency, gas impermeability and is easy to process. Its range extends from commodity [9,10,11,12,13] to biomedical [14,15,16,17,18] and pharmaceutical ones [19,20,21]. 

However, it has drawbacks that limit its use, such as brittleness, poor melt strength, low heat deflection temperature (HDT), narrow processing and low thermal stability. In some areas, it is limited by its hydrophobicity and degradation rates. The most common strategies to enhance the versatility of PLA and overcome these limitations is trough bulk or surface modifications. By following these strategies, the resulting copolymer has shown improved strength and toughness, in addition to controlled degradable and hydrophilic properties. It is also possible to obtain numerous new copolymers with different macromolecular architectures (linear, branch, star and dendron) [22,23,24,25].

In recent years many studies have been dedicated to the copolymerization of PLA with more hydrophilic monomers or polymers due to its high demand in the biomedicine industry. In this field, one important technology is 3D printing. PLA and its copolymers have proven to be easy to process and create complex 3D structures [26,27,28]. Copolymers such as poly(lactic acid-co-caprolactone) (PLCL) [29,30,31,32,33], poly(lactic acid-glycolic acid) (PLGA) [34,35,36] and poly(lactic acid-glutamic acid) (PLGM) [37,38,39] have been developed. The purpose of the present work is to synthetize a new copolymer of lactide and poly(propylene glycol) diglycidyl ether (PPGDGE) in order to improve the hydrophilic properties of PLA. We explore these properties through measuring the contact angle. Further, we aim to develop a biomaterial with an important building block that could help to tailor different structures and properties.

## 2. Materials and Methods

### 2.1. Materials

**Scheme 1:**
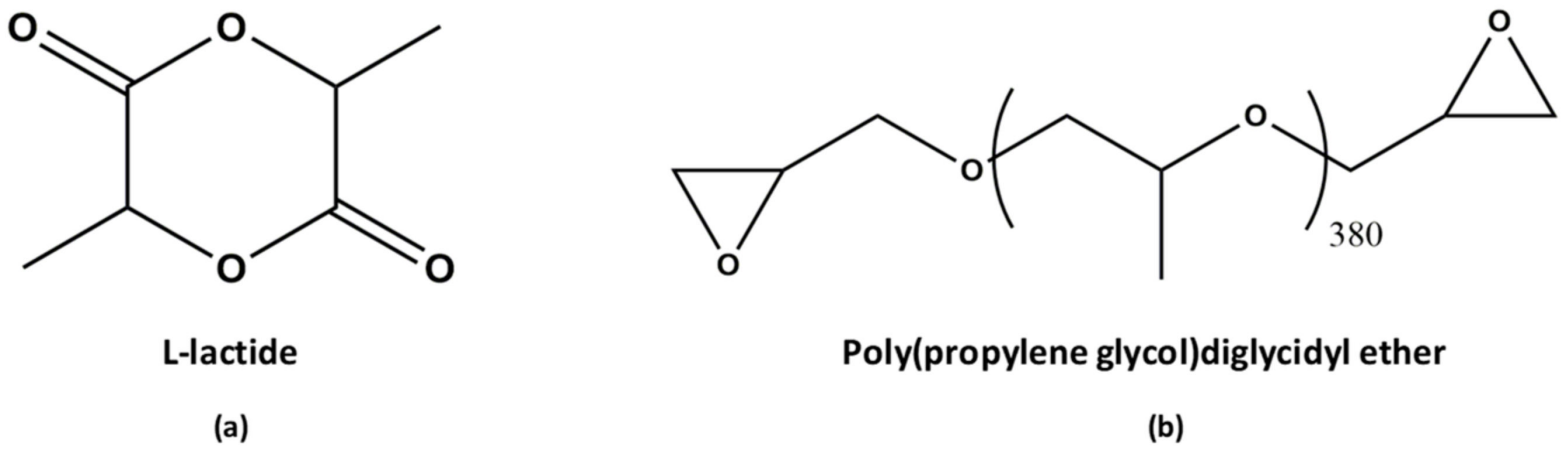
Structures of (**a**) l-Lactide and (**b**) Poly(propylene glycol)diglycidyl ether.

The l-lactide (98%, l-LA) (Scheme 1a) was purchased from Corbion (Amsterdam, The Netherlands), and was purified by recrystallization from toluene. Poly(propylene glycol) diglycidyl ether (PPGDGE_380_) (Scheme 1b), tin(II) 2-ethylhexanoate (Sn(Oct)_2_) and 1-dodecanol were purchased from Aldrich (Saint Louis, MS, USA); and were used as-received. Toluene, acetone and methanol were purchased from J.T. Baker (Mexico City, Mexico); reactive grade.

### 2.2. Copolymerization

The general process for the bulk copolymerization was as follows: We add the corresponding concentration of monomer ratio (l-Lactide/PPGDGE380) into a glass ampule, then, the catalyst (Sn(Oct)_2_) and the initiator (1-dodecanol) were added to the glass ampule and it was sealed. The vial was placed in a benchtop muffle furnace for a period of two hours. Once the reaction period was over, the resulting copolymers were kept in a vacuum chamber at 50 °C for four days to evaporate monomer residues. In the first set of experiments, different concentrations of monomer ratio were used (90/10, 80/20 and 70/30 wt % in feed). In the second set of experiments the monomer ratio concentration was kept constant and the catalyst/initiator ratio was varied. The cat/ini ratio was varied as follows: 0.01/0.1, 0.01/0.15, 0.01/0.2, 0.03/0.15, 0.05/0.15 wt %. The temperature used was 190 °C and the reaction period was set in two hours. For convenience and accuracy in weighing Sn(Oct)_2_ and 1-dodecanol were prepared as a 1 M and 3 M solution in toluene, respectively.

### 2.3. Characterization

The chemical structures of the copolymers were analyzed by Fourier transform infrared (FTIR) spectra and by ^1^H NMR spectrometry. To obtain the FTIR spectra to the investigated copolymers in the mid IR region (wavelength range: 400–4000 cm^−1^) an Excalibur FTS 3000 spectrometer provided by Mettler Toledo (Columbus, OH, USA); was used. The attenuated total reflectance (ATR) was used, utilizing a ZnSe crystal. The NMR spectroscopy was obtained from a Bruker of 500 MHz spectrometer (Billerica, MA, USA). Deuterated chloroform (CDCl_3_) was used as the solvent at room temperature. Thermal properties were obtained using a TA Q100 DSC calorimeter (TA Instruments-Waters LLC, Colonia Acacias, Mexico,). The device for the contact angle measurements was designed at the university, and consisted of a digital camera (Nikon, Melville, NY, USA), holder for the sample, a Gastight Glass Syringe to deposit a 12 μL drop of water, a UV light source that emits at 365 nm and a visible radiation lamp emitting at 580 nm. The angle was measured through a Java-based image processing program ImageJ, developed at the National Institutes of Health and the Laboratory for Optical and Computational Instrumentation, Wisconsin, United State. The calibration process was made using a glass slide and a water drop deposited in the surface, with any film. Copolymer films were prepared, to change the contact angle of the glass slide, by dissolving the samples into toluene and allowing it to evaporate in a desiccator to obtain a homogeneous film. The contact angle measurements were made using the static sessile drop method.

**Figure 1:**
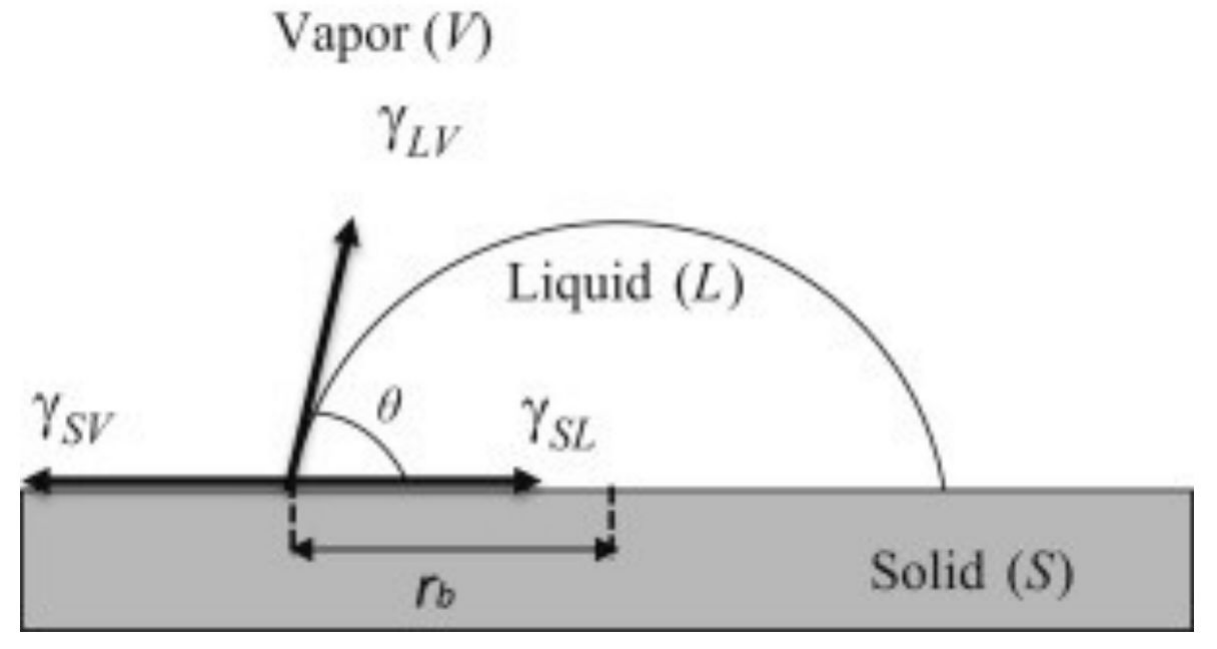
Static contact angle measurement with the sessile drop method.

This method is a static contact angle measurement method which consists of putting down a liquid drop on the solid plate surface we want to characterize, then measuring the contact angle made by the drop on this surface. Indeed, when a drop of liquid is deposited down on a solid surface, three phases occur: solid, liquid and gas (Figure 1).

The drop’s profile is being changed depending on the physicochemical characters of the solid surface, on the adhesion forces newly created at the interface solid/liquid and on the cohesion forces of the liquid. This change will affect the contact angle value revealing the surface state (hydrophobic or hydrophilic, rough or smooth, homogeneous or heterogeneous) and the different forces occurred are linked together according to Young’s Equation (1):

(1) $$\;\gamma_{s\nu }=\gamma_{sl}+\gamma_{l\nu }cos\theta \;$$

where *γ*_sv_, *γ*_sl_ and *γ*_lv_ represent the “surface tensions” of the interface solid/gas, solid/liquid and liquid/gas, respectively, and *θ* represents the contact angle.

## 3. Results

The PLA-PPGDGE copolymers were synthesized by the ring opening polymerization of successively added PPGDGE_Mn=380_ and l-lactide in the presence of 1-dodecanol, using tin(II) 2-ethylhexanoate as a catalyst. These copolymers differ greatly in appearance, from plastic like to gummy according to the monomer composition. The conversion of the polymerization was about 88–92%, with the residual monomer mainly l-lactide. These conversion measurements were based on monomer evaporation from the polymerized samples in a vacuum chamber.

**Figure 2:**
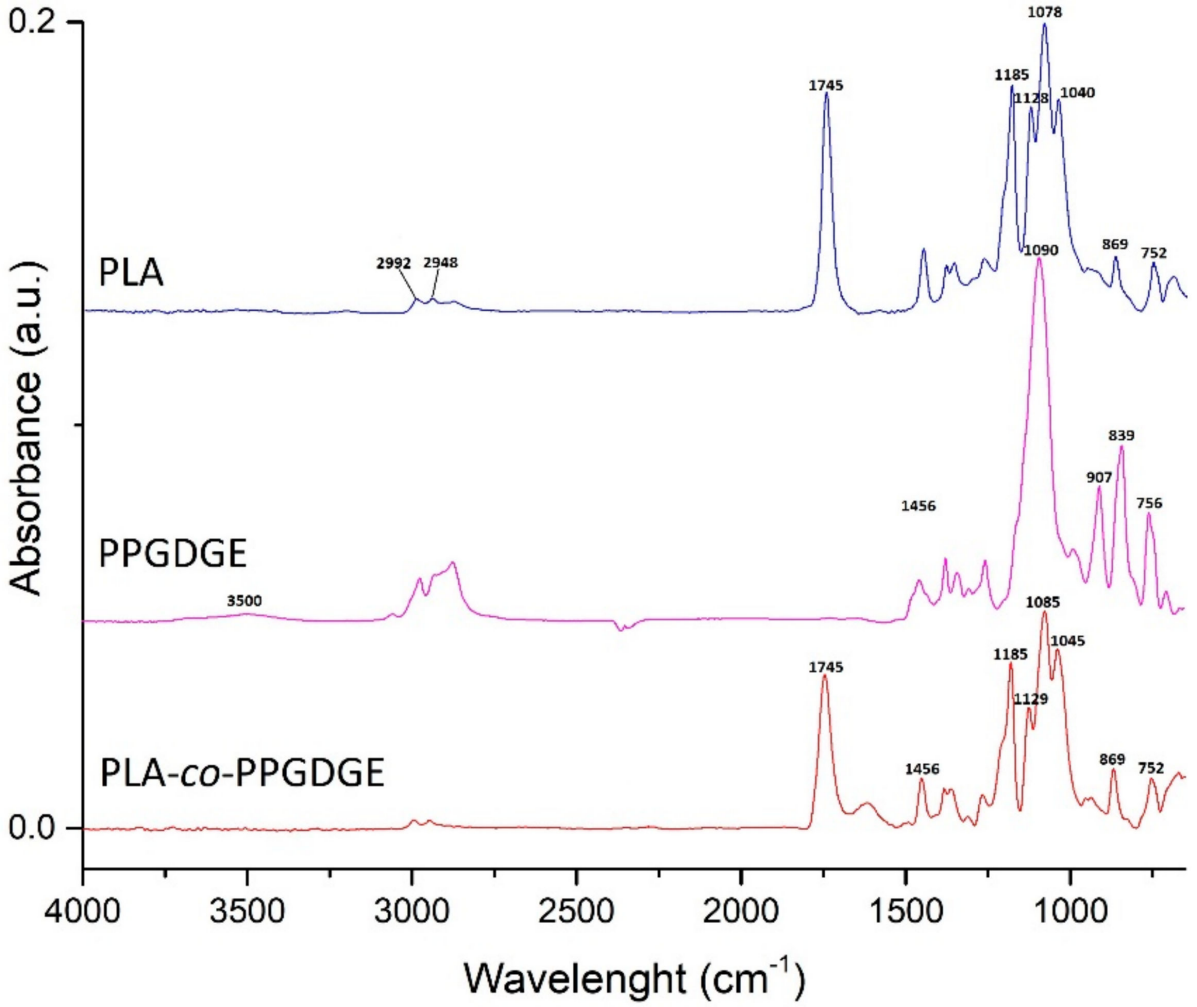
FTIR spectra of: PLA, PPGDGE, PLA and PLLA-co-PPGDGE. Copolymer ratio PLA/PPGDGE 80/20.

To examine the existence of the copolymer, FTIR experiments were performed and compared with those of the neat PLA and PPGDGE. Figure 2 shows the FTIR spectra of PLA, PPGDGE and the PLA-co-PPGDGE copolymer. For neat PLA, the peaks of interest are the C=O stretching at 1745 cm^−1^ and the O–C=O stretching at 1185 cm^−1^ which correspond to the characteristic ester bonds. It is possible to observe the C–O–C symmetric and symmetric band in the range of 1079–1181 cm^−1^, the symmetric bending of the C–H group at 1360 cm^−1^, the asymmetric bending of –CH_3_ at 1451 cm^−1^ and the symmetric and asymmetric bands to the –C–H stretching vibrations of CH_3_ groups in the side chain at 2992 and 2948 cm^−1^. For the plain PPGDGE there are three peaks observed at 907, 839 and 756 cm^−1^ corresponding to symmetric and asymmetric stretches of the epoxide ring [40] and the O-H stretching band at 3500 cm^−1^. The synthesis of the copolymer was confirmed by the disappearance of the epoxide ring bands in the copolymer spectra. It is possible for the formation of cross-linking and chain branching formation between diepoxy groups with both the end carboxyl and hydroxyl groups of the PLLA copolymer to produce primary ester and ether bonds, however this band was overlapped by the C–O–C asymmetric and symmetric band corresponding of PLA (1079–1181 cm^−1^). The spectra of the PLA base copolymer correspond to a copolymer with a monomer ratio of PLA/PPGDGE 80/20.

**Figure 3:**
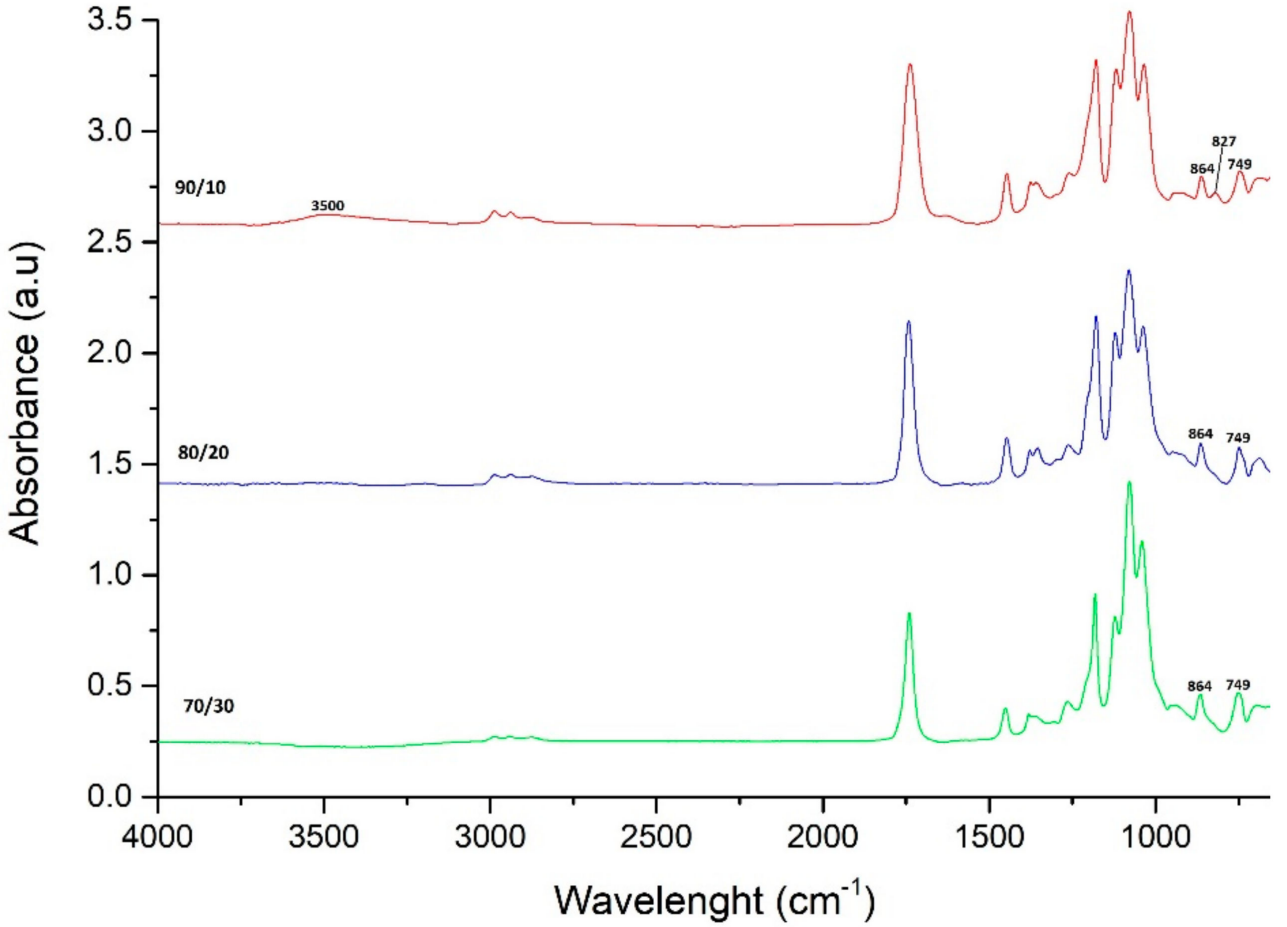
FTIR spectra of PLA-*co*-PPGDGE with different monomer ratio l-lactide/PPGDGE.

For the second set of experiments, it was necessary to keep the monomer ratio constant. The FTIR spectrums of the copolymers with different monomer ratios were analyzed (Figure 3). The spectra of the 90/10 (l-lactide/PPGDGE wt %) copolymer presents some unreacted PPGDGE because the symmetric and asymmetric stretches of the epoxide ring are present (907, 839, 756 cm^−1^) and the broad band of the O–H stretching of the hydroxyl groups at 3500 cm^−1^ can be seen. With 80/20 and 70/30 these bands completely disappear due to a complete reaction of the epoxy groups present in the PPGDGE. In appearance, the 70/30 (l-lactide/PPGDGE) copolymer was rubberier than the 80/20, because of the inclusion of more PPDGE chains into the copolymer. Based on this evidence, the monomer ratio chosen to carry on the second set of experiments was the 80/20 (l-lactide/PPGDGE).

**Figure 4:**
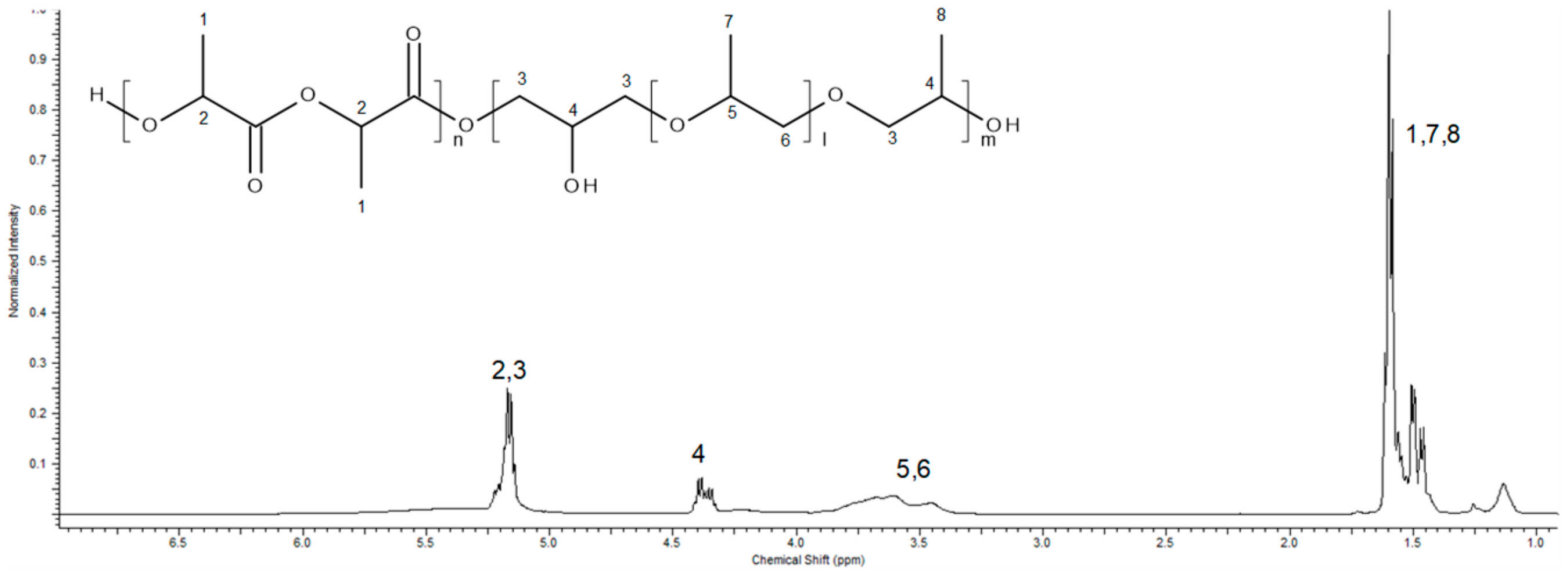
^1^H NMR spectrum of PLA-*co*-PPGDGE with monomer ratio 80/20.

The ^1^H NMR spectrum was taken in deuterated chloroform. It shows the typical signals of PLA and furthermore the PPGDGE signals (Figure 4). Signals at 5.1 ppm were assigned to methane protons (–CH) from the PLA block, the signals at 4.3 ppm belongs to methane protons of the carbon that is attached to the OH group that is formed when the epoxide ring is opened, the signals at 3.5 belonged to methylene protons (–CH_2_) from PPGDGE and, finally, the methyl protons (–CH_3_) from PPGDGE and PLA block were at 1.6–1.4 ppm.

Once the monomer ratio was set, the cat/ini ratio influence on the thermal behavior of the PLA based copolymers was studied. First the wt % of the catalyst was modified (0.01, 0.03, 0.05) and the wt % of the initiator was set in 0.15 wt %, after the wt % of the initiator was modified (0.1, 0.15, 0.2) and the catalyst wt % was set in 0.01%. All these modifications were made based on previous literature reports [41].

To evaluate the influence of the cat/ini ratio on the thermal behavior of copolymers differential scanning calorimetry (DSC) was used. All of the thermograms were obtained at a heating rate of 10 °C/min. The samples were initially heated to 115 °C, quenched to −60 °C and then heated from −60 °C to 115 °C at a heating rate of 10 °C/min. The first heating scan data were discarded because it included the previous thermal history. The results are listed in Table 1. The glass transition temperature (*T*_g_) for all of the materials was set as the temperature corresponding to the midpoint of the heat capacity increment.

**Figure 5:**
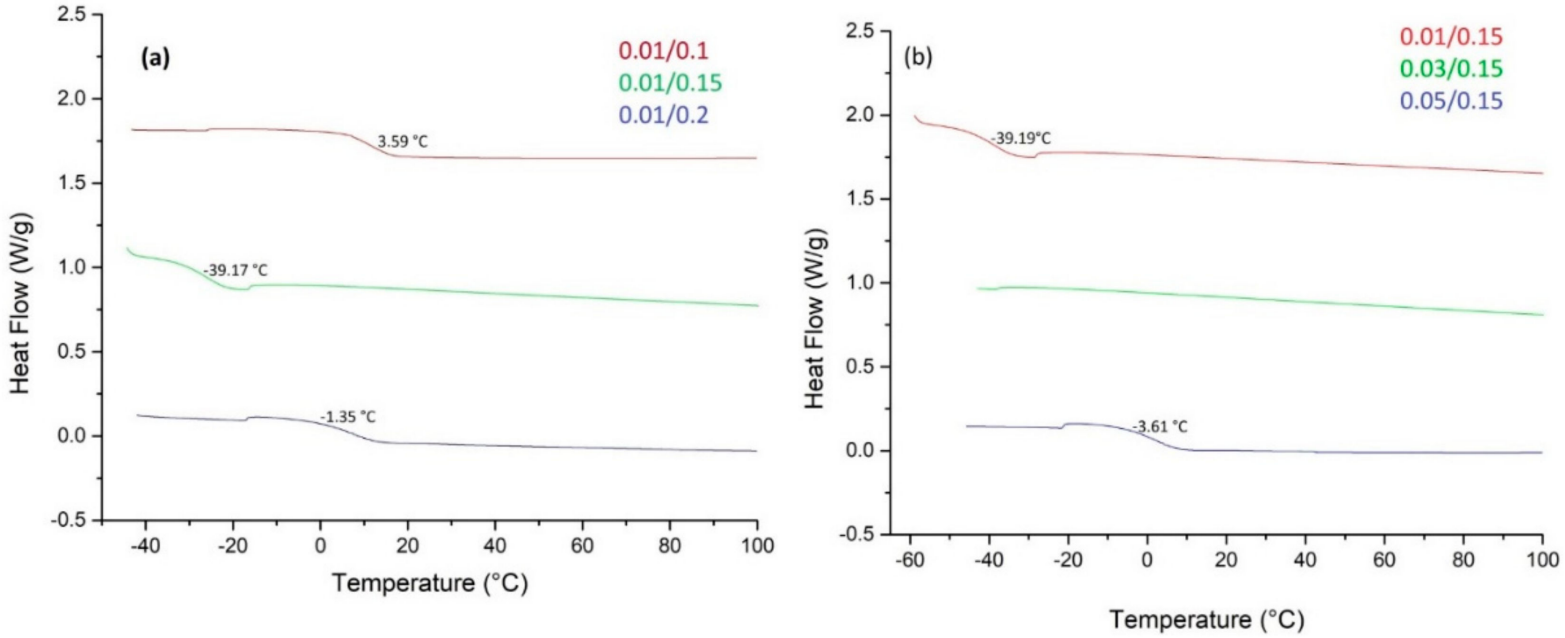
DSC thermograms of PLLA-*co*-PPGDE, monomer ratio 80/20 wt % with different cat/ini ratio. (**a**) Variation of 1-dodecanol. (**b**) Variation of Sn(Oct)_2_.

As observed, in Figure 5, at the thermogram (a) the amount of initiator (1-dodecanol) used to carry out the reaction was modified, it is observed that with concentrations around 0.15 wt % the formation of more chains of PPGDGE than PLA is being favored since the *T*g is around −39.2 °C. In the thermogram (b) the amount of Sn(Oct)_2_ used was varied and it is found that when a greater amount of catalyst is added, the *T*g can reach −3.61 °C, indicating in this case that raising the amount of Sn(Oct)_2_ would favor the formation of more PLA chains within the copolymer.

**Figure 6:**
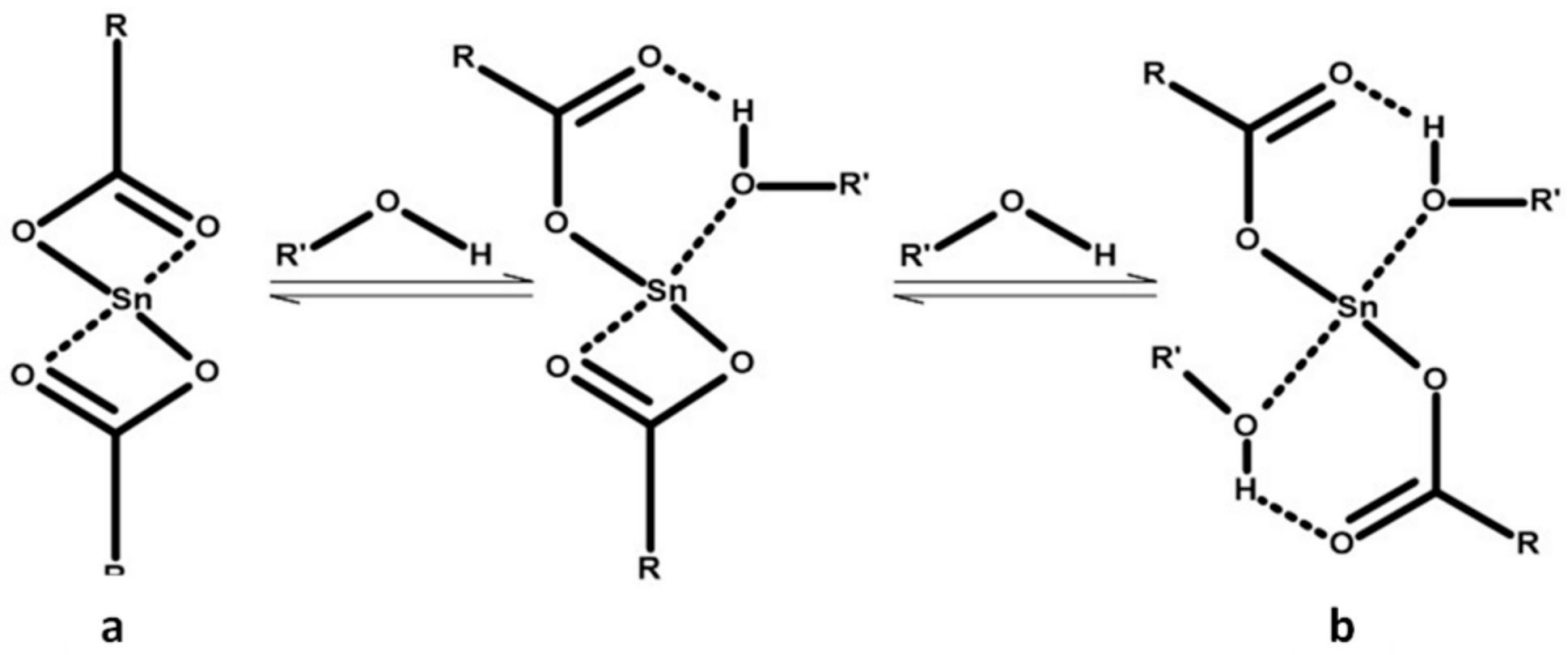
Mechanism of formation of the tin-alkoxide complex.

The data obtained indicates the effect of both the catalyst and initiator. For these copolymers, it was observed that there were ratios where the copolymer was formed mainly by chains of PPGDGE, and ratios where the copolymer chains were formed mostly by PLA. This could be related directly to the formation of the tin-alkoxide complex formed prior to initiation. According to different studies about the reaction mechanism [42,43,44,45] it is a consensus that before polymerization begins, a complex between the Sn(Oct)_2_ and the alcohol initiator will be formed. This complex is formed as shown in Figure 6, where the (a) Stannous octoate first reacts with two alcohol molecules to (b) form a tin alkoxide bond by ligand exchange.

Although the formation of this complex is very important for the final results, it must be kept in mind that this reaction is more complex because a copolymer was formed, and the presence of the other monomer played an important role. In addition, the stannous octoate hydrolyzes in the presence of impurities, like water, and is difficult to remove from the monomers. The 2-ethylhexanoic acid from the chemically polluted stannous octoate was also an issue.

**Figure 7:**
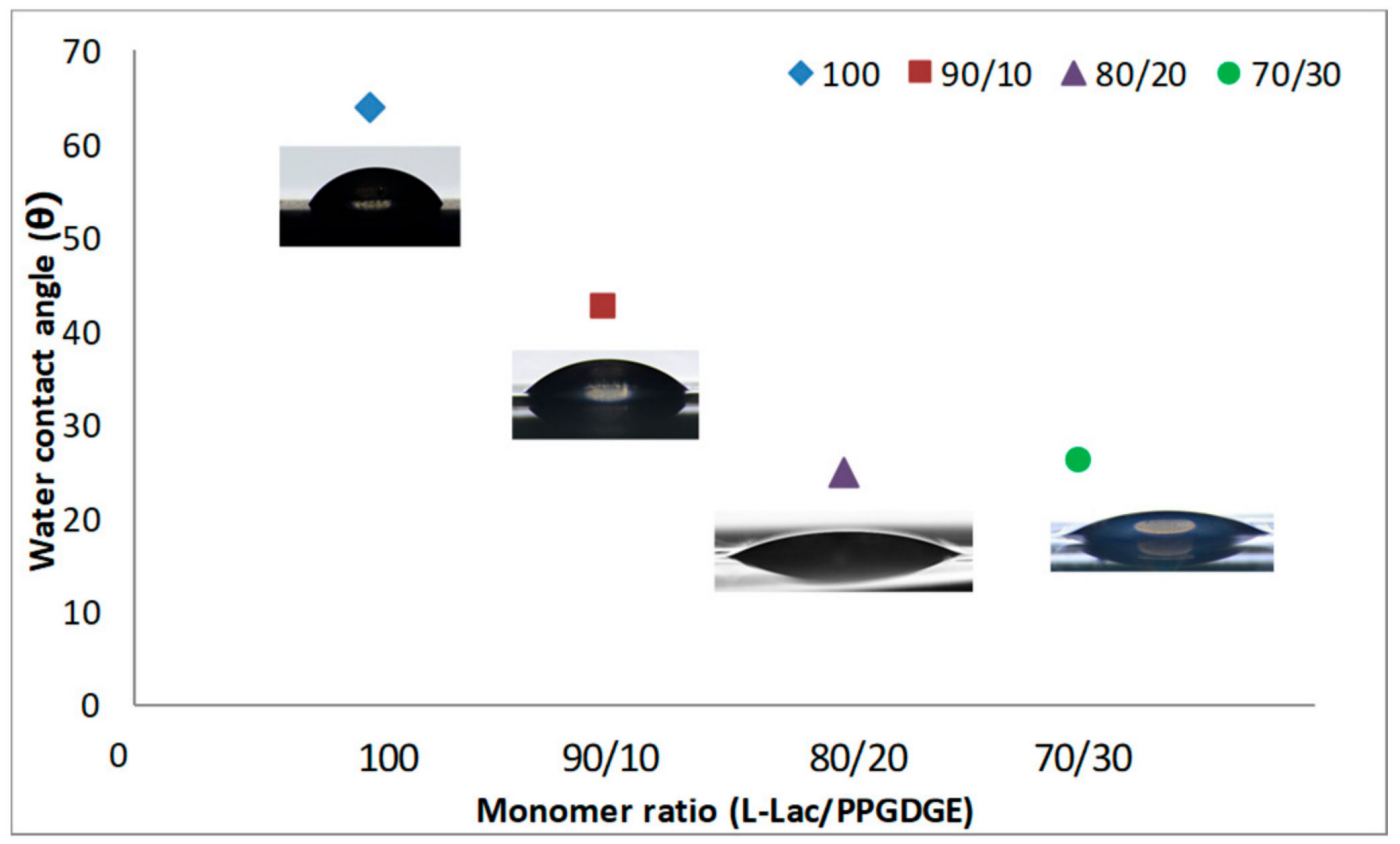
Contact angle of PLA-co-PPGDGE by varying monomer ratio. cat/ini ratio 0.01/0.15 wt %.

Finally, the wettability properties of the synthesized copolymers were evaluated using measures of contact angle. As seen in Figure 7, a decrease in contact angle measurements were exhibited in all the copolymers compared with neat PLA. An obvious hydrophilic improvement with the increasing constitution of the PPGDE in the copolymer is observed. It is known that the PPGDGE is a polymer with alternate hydrophobic and hydrophilic groups in molecular chains, but in this case the hydrophilic parts of the PPGDGE had a greater influence within the copolymer. The hydrophilicity modifications are confirmed by the rapid contact angle decreasing in the first 10 s, and it serves as an evidence for the copolymer hydrophilic improvement when PPGDGE is introduced. When 30% of this monomer was added, the contact angle decreased almost 38° in comparison with pristine PLA. As can be seen, the increases of PPGDGE in the monomer ratio decreases the contact angle, this is in accordance with what was previously described in the literature. Alli et.al., found a similar behavior [46], as they included more PPG inside the study copolymer, the contact angle was decreasing.

**Figure 8:**
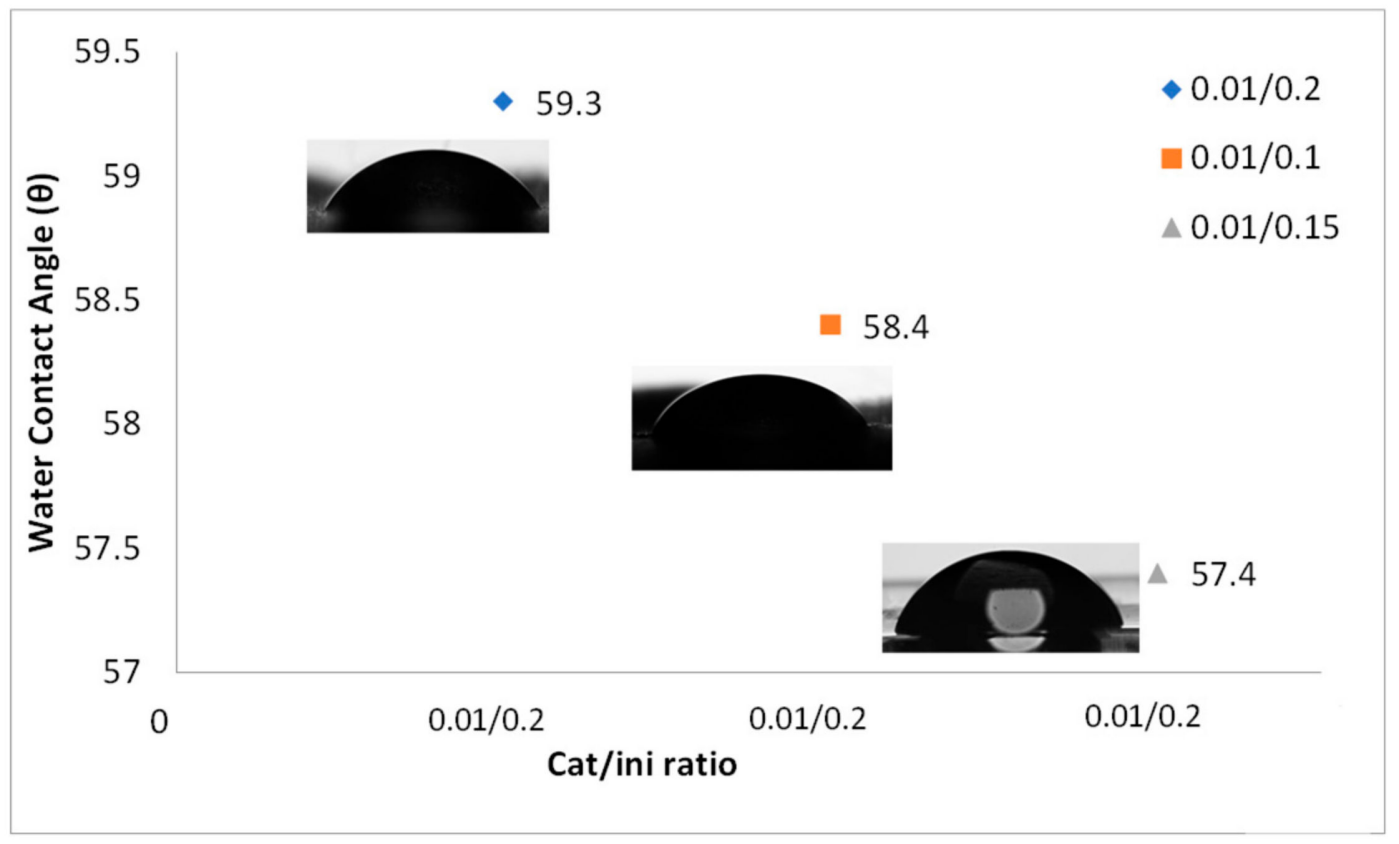
Contact angle of PLA-co-PPGDGE by varying cat/ini ratio. Copolymer 80/20 (l-lactide/PPGDGE).

The change in the cat/ini ratio does not have a strong influence in the contact angle (Figure 8), however as the cat/ini rate increases, the contact angle decreases. But this decrement is very slight; the change is about 1°. As analyzed previously by Xiao et al. [47] for the polymerization of 2-ethoxy-2-oxo-1,3,2-dioxaphospholane initiated by Sn(Oct)_2_, further increase of Sn(Oct)_2_ or dodecanol does not provide any more active species. That is why we cannot see a drastic change in the contact angle when we use those cat/ini rates.

All the results of the contact angle measurements agreed with the glass transition data obtained through DSC. For example, the copolymer with monomer ratio 80/20 and cat/ini ratio of 0.01/0.15 wt % was the one with the lowest glass transition temperature (*T*g). The *T*g was observed at −39.15 °C and the contact angle was 24.8°, confirming that this copolymer had more PPGDGE chains than PLA chains, which made the material more hydrophilic.

## 4. Conclusions

It was concluded that it is possible to tailor the hydrophilic properties of the copolymers of PLA and PPGDGE through the variation of monomer ratios, depending of the properties of interest. The inclusion of the PPGDGE into the PLA also modified the mechanical properties, as it was observed through the *T*g, less rigid copolymers were obtained in comparison with pure PLA. The medical importance of these kind of materials make them very important, so additional knowledge about their behavior may enable increased usage.

## Figures and Tables

**Table 1:** Glass transition temperatures of PLA copolymers with different cat/ini ratio.

Sample	Cat/Ini (wt %)	*T*_g_ (°C)
1	0.01/0.1	3.59
2	0.01/0.15	−39.19
3	0.01/0.2	−1.35
4	0.03/0.15	------
5	0.05/0.15	-3.61
